# Integrating Surveillance and Stakeholder Insights to Predict Influenza Epidemics: A Bayesian Network Study in Queensland, Australia

**DOI:** 10.3390/ijerph23010069

**Published:** 2026-01-01

**Authors:** Oz Sahin, Hai Phung, Andrea Standke, Mohana Rajmokan, Alex Raulli, Amy York, Patricia Lee

**Affiliations:** 1 School of Engineering and Built Environment, Griffith University, Gold Coast, QLD 4222, Australia; o.sahin@griffith.edu.au; 2 School of Public Health, The University of Queensland, Brisbane, QLD 4066, Australia; 3 School of Medicine and Dentistry, Griffith University, Gold Coast, QLD 4222, Australia; hai.n.phung@griffith.edu.au (H.P.); amy.york@griffithuni.edu.au (A.Y.); 4 Clem Jones Centre for Neurobiology and Stem Cell Research (CJCNSCR), Griffith University, Gold Coast, QLD 4222, Australia; a.standke@griffith.edu.au; 5 Institute for Biomedicine and Glycomics, Griffith University, Gold Coast, QLD 4222, Australia; 6 Communicable Disease Epidemiology, Public Health Intelligence Branch, Population Health Division, Queensland Health, Brisbane, QLD 4006, Australia; mohana.rajmokan@health.qld.gov.au; 7 Epidemiology Section, Australian Capital Territory (ACT) Health, ACT Government, Canberra, ACT 2606, Australia; alex.raulli@act.gov.au; 8 Department of Medical Research, China Medical University Hospital, Taichung City 404, Taiwan

**Keywords:** bayesian network modelling, public health decision support, influenza epidemics forecasting, scenario analysis, climate and influenza dynamics

## Abstract

**Highlights:**

**Public health relevance—How does this work relate to a public health issue?**

- The study addresses the ongoing global public health challenge of seasonal influenza by using a Bayesian Network (BN) model to examine how surveillance data, population characteristics, and contextual risk factors interact to influence epidemic occurrence in Queensland.
- The BN framework captures the complexity and uncertainty inherent in influenza transmission and epidemic emergence, potentially contributing to the planning and preparedness for infectious disease outbreaks.

**Public health significance—Why is this work of significance to public health?**

- The study demonstrates that a BN-based approach, integrating surveillance data, environmental/climatic indicators with expert and stakeholder knowledge, enhances epidemic risk estimation and response.
- The findings provide a transparent and interpretable modelling framework that quantifies uncertainty and supports evi-dence-informed decision-making in influenza preparedness.

**Public health implications—What are the key implications or messages for practitioners, policy makers and/or researchers in public health?**

- Public health practitioners can apply the BN model to explore “what-if” scenarios, identify high-risk regions and conditions, and guide targeted vaccination, travel risk management, and timely control measures.
- Policymakers and researchers may use the BN framework to support adaptive preparedness planning, evaluate intervention strategies under uncertainty, and extend the model to other climate-sensitive or emerging infectious diseases.

**Abstract:**

Seasonal influenza continues to pose a substantial and recurrent public health challenge in Queensland, driven by annual variability in transmission and uncertainty in climatic, demographic, and behavioural determinants. Predictive modelling is constrained by data limitations and parameter uncertainty. In response, this study developed a Bayesian network (BN) model to estimate the probability of influenza epidemics in Queensland, Australia. The model integrated diverse inputs, including international and local influenza surveillance data, demographic health statistics, and expert and stakeholder insights to capture the complex multifactorial causal relationships underlying epidemic risk. Scenario-based simulations revealed that Southeast Asian viral origin, severe global influenza seasons, peak season timing, increasing international travel, absence of control measures, and low immunisation rates substantially elevate the likelihood of influenza epidemics. Southeast Queensland was identified as particularly vulnerable under high-risk conditions. Model evaluation demonstrated good discriminative performance (AUC = 0.6974, accuracy = 70%) with appropriate uncertainty quantification through credible intervals and sensitivity analysis. Its modular design and capacity for integrating various data sources make it a practical decision-making support tool for public health preparedness and responding to evolving climatic and epidemiological conditions.

## 1. Introduction

Seasonal influenza is an acute respiratory infection caused by influenza A and B viruses, producing annual epidemics and occasionally pandemics [1]. Although many infections are mild and self-limiting, the high transmissibility of influenza viruses facilitates extensive community transmission, resulting in significant work and school absences and associated productivity losses [2]. However, seasonal influenza epidemics can also lead to severe illness and mortality. The World Health Organization (WHO) estimates that seasonal influenza is responsible for approximately 3–5 million cases of severe illness and 290,000–650,000 respiratory deaths worldwide annually [3].

In temperate regions, seasonal influenza is driven by cold and dry climatic conditions, which commonly coincide with the winter months [4]. Several experimental and epidemiological studies suggest that low absolute humidity, produced by cold temperatures and indoor heating, is one of the most significant predictors of influenza transmission and seasonality in these regions [5,6,7]. In contrast, peak influenza incidence occurs during rainy conditions in tropical and subtropical climates [8]. Although airborne transmission is reduced in warmer conditions, the wet conditions increase the survival and deposition of viral droplets on fomites, therefore facilitating increased contact transmission [9]. This is further amplified by increased indoor crowding during rainy weather [10]. However, compared to temperate regions, influenza seasonality in subtropical and tropical regions is less pronounced and may exhibit multiple peaks or demonstrate no clear seasonal pattern [11]. Other meteorological factors that impact influenza transmission include wind speeds, which can cause aerosols produced by an infected individual to travel further, and reduced solar radiation, which potentially impairs immune function through decreased vitamin D production [8].

Social contact between susceptible and infected hosts is another important aspect in influenza virus transmission, and measures to reduce contact rates are common interventions in seasonal influenza epidemics [12,13]. For example, scheduled school holidays can substantially reduce transmission, with one study in Alberta, Canada reporting a more than 50% reduction in transmission rates among school children during scheduled holidays in the 2009 H1N1 influenza pandemic [14]. Furthermore, patterns in human movement may explain the spatial characteristics of seasonal influenza epidemics. At a regional scale, influenza virus circulation exhibits a localised, radial spatial structure that reflects patterns of human mobility, including work commutes [15]. However, over longer distances, air travel may be an important mode of dissemination due to the close proximity of travellers and the better connection between distant regions [16]. One study found that 3% of susceptible passengers developed influenza-like illness (ILI) during flights in the 2009 H1N1 pandemic [17].

Influenza surveillance data, together with information regarding transmission dynamics, can be used to create predictive models that provide valuable insights into the magnitude, timing, and spatial distribution of outbreaks [18]. A range of modelling approaches have been applied to influenza forecasting in Australia and internationally, each with distinct strengths and limitations. Time-series models (e.g., Auto Regressive Integrated Moving Average (ARIMA) and exponential smoothing (ETS)) excel at short-term forecasting based on historical patterns but are limited in their ability to incorporate causal mechanisms, diverse data types, and external factors beyond temporal trends [19,20]. Mechanistic transmission models (e.g., SEIR (susceptible-exposed-infected-recovered) model and agent-based models) provide detailed representations of disease dynamics and can simulate intervention effects, but require precise parameter estimation, extensive computational resources, and may be challenging to adapt for real-time decision support [21,22]. Fractional-order epidemic models, which improve representation of memory-dependent transmission processes, were also explored in a study; however, these approaches still require continuous, high-resolution temporal data and precise parameter estimates [23].

Bayesian networks (BNs) offer a complementary modelling approach particularly suited to settings with uncertainty, incomplete data, and complex causal interactions. As probabilistic graphical models, BNs represent variables and their conditional dependencies using directed acyclic graphs (DAGs), enabling transparent visualisation of causal relationships [24]. BNs can integrate heterogeneous data sources, including surveillance data, climatic variables, demographic factors, and expert knowledge, within a unified probabilistic framework [25]. BNs can also address the limitations of purely qualitative methods by systematically modelling complex relationships and uncertainties in decision-making [26]. Furthermore, BNs naturally accommodate missing data, allow scenario-based “what-if” analysis, and produce interpretable probabilistic outputs suitable for communication to decision-makers [27].

Despite these advantages, BN applications for influenza prediction in Australia remain limited, and few studies have combined international and local surveillance, climate variables, and expert insight within a single probabilistic model [28,29]. The performance of these models also varies by location due to reporting artefacts and differences in demographic, environmental, and social factors. In addition, influenza seasons can differ widely based on population immunity, vaccination coverage, and the emergence of genetic variants. Therefore, these factors need to be considered in predictive models [18].

This study addresses this gap by developing a Bayesian network model to estimate influenza epidemic probability in Queensland, Australia, across multiple climatic regions. The model integrates international and local surveillance data, demographic and climatic variables, and expert-elicited probability estimates to capture multifactorial interactions. Scenario analyses are used to explore the impact of extreme or high-risk conditions, while sensitivity analyses identify the most influential determinants of epidemic risk. Model performance is evaluated through retrospective validation against documented influenza epidemics.

Specific objectives of this study are to:

1. Develop a probabilistic BN model integrating surveillance, climatic, demographic, and expert-elicited data;
2. Quantify conditional dependencies among key epidemic determinants;
3. Evaluate model performance using retrospective surveillance data from Queensland;
4. Conduct scenario analyses to examine how variable changes influence epidemic probability;
5. Identify influential determinants through sensitivity analyses; and
6. Demonstrate the model’s potential as an early-warning decision-support tool for infectious disease prevention and outbreak control.

By integrating heterogeneous data sources within a transparent probabilistic framework, this study contributes to the growing application of Bayesian methods for epidemic preparedness, offering a flexible and interpretable tool for public health decision-making in Queensland and similar settings.

## 2. Materials and Methods

### 2.1. Data Sources

This study utilised real observational data (not simulated) from multiple sources covering the period 2011–2020:

- Surveillance data: The weekly number of influenza notifications, including laboratory-confirmed influenza cases (by Queensland Hospital and Health Services), was obtained from Queensland Health’s influenza surveillance reports [30]. Additional surveillance data was sourced from WHO FluNet to inform viral circulation patterns in Australia over time, and this was used as a proxy to estimate Queensland patterns [31].
- Climate data: Daily temperature (°C), relative humidity (%), and rainfall (mm) were obtained from the Australian Bureau of Meteorology for representative stations in Southeast Queensland (Brisbane), Central Queensland (Rockhampton), and North Queensland (Cairns) [32].
- Demographic data: Population estimates by age group, regional population density, and international arrivals data were obtained from the Australian Bureau of Statistics [33,34,35].
- Epidemic definition: Based on Queensland Health’s notifiable conditions surveillance practice, we define an influenza epidemic as “the accumulated notifications of an observed season were exceeding the average notifications in 2011–2015 (baseline) by 10% in the same location” [36].
- Data structure: Weekly observations from all data sources were aligned by calendar week and region, producing 520 multi-variable records (52 weeks × 10 years) for model development and validation. Three documented prominent epidemic periods (2015, 2017, and 2019) were identified from surveillance reports and used for retrospective validation.

### 2.2. Bayesian Network Model Development

A Bayesian network (BN) consists of nodes (variables), directed edges representing conditional dependencies, and conditional probability tables (CPTs) quantifying these dependencies. The development process followed established BN modelling frameworks described in the literature [25,37,38,39]. In these frameworks, each node in the network represents a variable, and edges (arrows) between nodes indicate probabilistic dependencies. The relationships are defined by CPTs, which quantify how the probability of a variable changes given the values of its parent variables. The graphical nature of BNs therefore enables them to be understood by non-technical users [40]. The development of the BN model procedure used in this study was adapted from [37,41], and involved several steps: (1) Defining objectives, which involved establishing the aims of the model and its intended users; (2) Conceptual modelling, whereby an initial diagram was created demonstrating key influences; (3) Network construction, which involved defining the states of each variable and specifying model parameters; (4) Model evaluation, where model performance was assessed; and (5) Scenario analysis, where various scenarios were tested to assess potential outcomes.

Variable selection was guided by the literature review, surveillance system requirements, and iterative stakeholder consultations. Three workshops involving epidemiologists, infectious disease physicians, climate scientists, and public health practitioners refined model boundaries, node definitions, and causal assumptions. The Research Team first constructed the model components based on the influenza literature [4,10,42,43,44]. In the stakeholder engagement workshops and consultation sessions, the modeller (the first author, OS) worked collaboratively with the experts/stakeholders to further refine the model structure and variables, considering the Australian/Queensland context. Existing local data was also consulted to determine the distributions of certain demographic and environmental variables. For instance, the ‘Influenza in Queensland 2013–2018’ report was used to determine the gender and age distributions of influenza cases [45]. Continuous variables were discretised using thresholds supported by empirical research and validated with experts:

- Temperature: <5 °C (rare in Queensland, included for completeness), 5–20 °C (associated with greater viral stability), >20 °C (less favourable for sustained transmission) [7,8].
- Humidity: Low (<40%), Moderate (40–70%), High (>70%) based on absolute humidity thresholds associated with influenza transmission [46,47].
- Rainfall: Low (<50 mm/month), Moderate (50–150 mm), High (>150 mm) based on Queensland seasonal patterns.
- Age groups: <5, 5–65, >65 years to align with established risk categories used in Queensland influenza surveillance and vaccination prioritisation programs [45].
- Seasons: Mar–May, Jun–Aug, Sep–Nov, Dec–Feb. Seasons were included as a separate variable to represent southern hemisphere seasonality independent of climate variable discretisation.

The Research Team was also interested in the potential impact of climate change on the key climatic factors influencing influenza seasonality patterns. In order to evaluate this, the RCP (representative concentration pathway) 2.6 temperature change scenario was used [48]. The variable definitions and their data sources for the model are detailed in Supplementary Table S1. Artificial intelligence (AI) tools (OpenAI ChatGPT v5 and Claude AI) were used during model development to support preliminary literature identification and variable categorisation. Each tool was applied independently to identify reputable studies on multifactorial influenza models and to summarise viral characteristics, demographic factors, and environmental/climatic variables. Outputs were compared to identify common variable categories influencing influenza transmission, which informed subsequent variable selection for the Bayesian network (BN) model.

#### 2.2.1. Model Structure Development

The BN structure (node connections) was determined through an iterative process:

1. Literature review: The initial model structure was based on established epidemiological relationships from the influenza transmission literature [6,13,36,37,38];
2. Expert workshops: Three half-day workshops with stakeholders (n = 5: epidemiologists, infectious disease physicians, public health officials, climate scientists) were utilised to validate proposed relationships, identify additional relevant variables specific to the Queensland context, and ensure the model’s scope aligned with decision-maker needs;
3. Structural validation: Preliminary structure validation occurred through expert review for causal plausibility and alignment with domain knowledge; and
4. Iterative refinement: The model structure was revised based on predictive performance, stakeholder feedback on interpretability, and computational feasibility.

The final structure therefore balances empirical evidence, expert knowledge, practical data availability, and public health planning needs.

#### 2.2.2. Conditional Probability Table Construction Method

Conditional probability tables (CPTs) were populated using a structured, multi-source approach, which involved:

- Expert panel: Five experts participated in CPT elicitation: two epidemiologists (15+ years of experience in influenza surveillance), two infectious disease physicians (10+ years of clinical experience), and one climate scientist (25+ years of experience in climate-health interactions). Experts were selected based on their experience/involvement in influenza surveillance and public health response.
- Elicitation method: A consensus technique was employed over two rounds of workshops [49]. During round one, experts independently assessed “best case”, “typical case”, and “worst case” scenarios to anchor probability distributions for each CPT. Then, in round two, the experts revised estimates based on group responses and evidence. Any divergences exceeding 20% triggered facilitated discussion, and probability assignments were cross-checked for monotonicity and plausibility using the scientific literature.
- Consistency checks: The consistency checks comprised of three components, (1) Logical coherence, whereby CPTs were assessed for expected monotonicity (e.g., higher temperature should not arbitrarily increase epidemic probability); (2) The literature validation, which involved comparing probability distributions against empirical effect sizes from published studies where available; and (3) Sensitivity testing, where extreme CPT values were tested to ensure they produced logically consistent network outputs.

Once the BN and CPTs were created, subsequent workshops were completed to undertake scenario analysis, including both forward and backward network analyses. All modelling, scenario analyses, and sensitivity assessments were performed using GeNIe 5.0 [50].

#### 2.2.3. Scenario Analysis and Evidence Setting

Scenario analyses involved setting “hard evidence” (100% probability) on selected nodes to explore extreme or policy-relevant conditions. This approach follows standard BN scenario analysis methods and is equivalent to estimating conditional probabilities under specified conditions (e.g., P(Epidemic|Severe global season, Peak winter, SEQ)) [27].

### 2.3. BN Model Performance and Evaluation

The evaluation of a Bayesian network model requires an iterative process involving multiple stages to ensure its accuracy, robustness, and alignment with domain realities. BN model evaluation in this study involved the following components:

- Model structure and CPTs: Developed through expert workshops using domain knowledge from the influenza transmission literature, Queensland surveillance patterns, and expert consensus.
- Historical data usage: Informed prior probabilities for observable nodes (e.g., seasonal distributions, regional patterns) and provided context for expert elicitation (typical vs. extreme scenarios) but did not train model parameters in a machine learning sense.
- Testing procedure: Since the BN model was developed based on expert knowledge rather than a data-driven (machine learning) model, GeNIe’s “Test Only” validation approach was used, which is specifically designed for expert knowledge-based models. This method is primarily used to assess the predictive performance of a BN model on a set of real-world observations.
- Output metrics: (1) Accuracy, defined as the percentage of records for which the model correctly predicted the state of the Class Node; (2) Confusion matrix, a table containing the breakdown of correct and incorrect predictions for each state of the Class Node; (3) ROC (Receiver Operating Characteristic) curves and corresponding AUC (Area Under the Curve) values, measuring the performance of the classifier across different decision thresholds; and (4) Sensitivity analysis, quantifying the influence of each variable on the probability of an influenza epidemic using Tornado diagrams. Relative influence values were also computed by measuring the maximum shift in posterior probability when each variable was fixed at its extreme states.

## 3. Results

### 3.1. Bayesian Network for Influenza Epidemic Risk Assessment in Queensland

**Figure 1:**
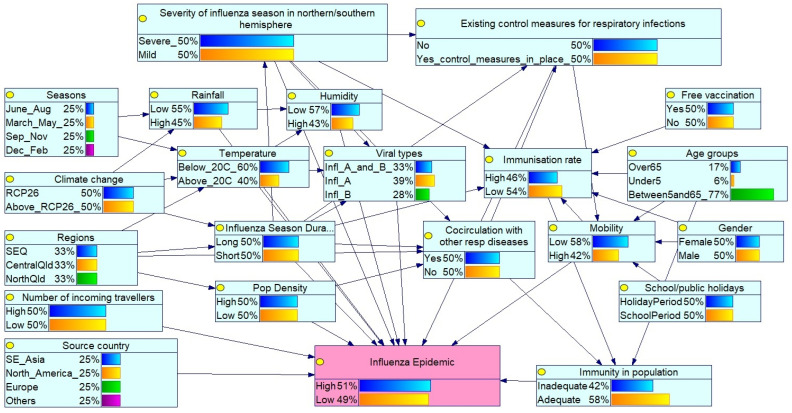
Bayesian network for influenza epidemic risk assessment in Queensland (baseline scenario). Light blue: indicates the influencing factors; Pink: indicates the outcome variable (Influenza Epidemic).

The Bayesian network (Figure 1) models the probability of an influenza epidemic in Queensland (Qld), Australia, based on a set of interdependent factors. The network’s structure, visualised as a directed acyclic graph, illustrates the probabilistic relationships among variables. The arrows in the network depict the conditional dependencies, showing how the change in one variable influences the likelihood of others.

The central node, “Influenza Epidemic” (in pink), represents the outcome of interest with binary states: “High” and “Low”. Its probability is influenced by a network of parent nodes representing various epidemiological, environmental, and demographic factors (in light blue). These are categorised as follows:

- Global influenza season: Severity in the northern/southern hemispheres reflects circulating virus types and virulence.
- Public health interventions: Existing control measures such as mask mandates, social distancing during the COVID-19 pandemic, travel restrictions, and influenza vaccination coverage affect transmission. Free vaccine availability influences the immunisation rates within the population, while increased incoming travellers reflect population mobility.
- Seasonality patterns: Seasons (Mar–May, Jun–Aug, Sep–Nov, Dec–Feb) reflect southern hemisphere influenza trends, with peak activity typically in winter (Jun–Aug). Influenza season duration (long or short) also modulates epidemic potential.
- Environmental/climatic conditions: Temperature, humidity and rainfall affect virus survival and transmission. Climate change, represented by RCP 2.6 and ≥RCP 2.6 scenarios, may alter seasonal dynamics.
- Geographical and demographic factors: Regions (Southeast Qld (SEQ), Central Qld, North Qld) capture climatic and population density variations. The selected Queensland regions represent different climatic zones according to their latitudinal gradients (temperate, subtropical, and tropical). The SEQ region is the most populous region in Queensland. The source country (SE_Asia (South East Asia), North America, Europe and Others) of the viral strain is also considered.
- Virological and host factors: Virus types (influenza A/B), age groups (under 5, 5–65, over 65), gender, and co-circulating respiratory pathogens (e.g., COVID-19 and respiratory syncytial virus (RSV)) influence susceptibility and diagnosis. Mobility and school/public holidays also affect transmission dynamics.
- Population immunity: Determined by age, vaccination status, and prior exposure to influenza or other respiratory infections.

Figure 1 presents a baseline scenario with a moderate influenza epidemic probability (50.7% high, 49.3% low) based on prior probabilities: equal seasonal distribution (25% per season), moderate climate change impact (50% ≤ RCP 2.6, 50% > RCP 2.6), and equal regional representation (33.3% each for SEQ, Central and North Qld). The baseline scenario represents prior probabilities before any specific evidence is entered. Prior probabilities for each node were derived from: (1) historical frequency distributions from 2011–2020 Queensland surveillance data for variables with available data (e.g., seasons, regions); (2) literature-derived probabilities for variables without local data (e.g., global influenza severity based on WHO global surveillance); and (3) expert consensus from the elicitation process for relationships with limited empirical data. The baseline thus represents “expected conditions” under historical patterns and serves as the reference point for scenario comparisons.

### 3.2. Scenario Analysis Results

To assess the impact of specific conditions on the probability of a high influenza epidemic in Queensland, a series of scenarios was developed. In each scenario, selected node probabilities were set to 100% to simulate extreme or high-risk situations. These scenarios illustrate how combinations of epidemiological, environmental, and behavioural factors influence epidemic potential within the Bayesian network (BN) framework.

#### Scenario 1: Severe Peak Season Epidemic in SEQ with High Circulation and No Controls

**Figure 2:**
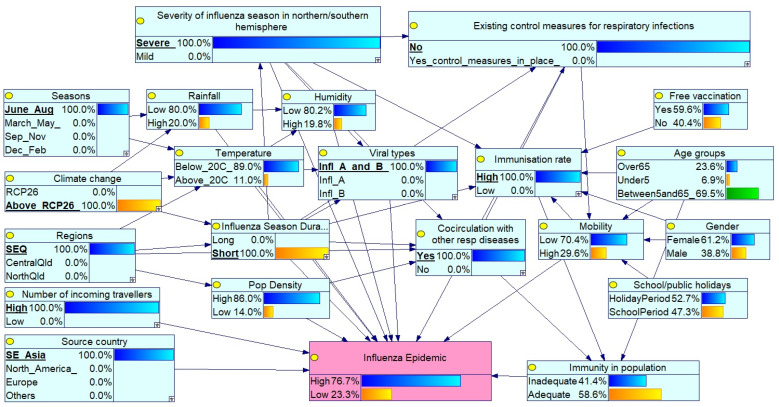
Impact of adjusted node probabilities on the influenza epidemic. Light blue: indicates the influencing factors; Pink: indicates the outcome variable (Influenza Epidemic).

This scenario (Figure 2) simulates a severe global influenza season coinciding with peak winter months (June–August) in Southeast Queensland (SEQ). All control measures are absent, and co-circulation of influenza A and B with other respiratory diseases (e.g., COVID-19 and RSV) is present. Despite a high immunisation rate, the epidemic risk is elevated due to increased population mobility, with a high volume of incoming travellers and virus introduction from Southeast Asia. The probability of a high influenza epidemic is assessed under these extreme conditions by setting relevant node probabilities to 100%.

### 3.3. Impact of Node Probability Changes on Epidemic Risk

To simulate an extreme scenario, selected node probabilities were set to 100%, representing high-risk conditions. These included:

- Severity of influenza season in northern/southern hemisphere: set to Severe, indicating widespread circulation of virulent strains.
- Existing control measures for respiratory infections: set to No, reflecting the absence of interventions such as mask mandates or social distancing.
- Seasons: set to June–August, aligning with peak influenza activity in the southern hemisphere (including Queensland).
- Climate change: set to above RCP 2.6, representing significant long-term climate shifts potentially affecting disease patterns.
- Regions: set to SEQ, focused on the SEQ Region.
- Co-circulation of respiratory pathogens: set to Yes, indicating simultaneous circulation of influenza A and B, along with other respiratory viruses (e.g., COVID-19 and RSV).
- Source country: set to Southeast Asia, suggesting a potential source of the influenza virus.
- Number of incoming travellers: set to High, reflecting elevated population mobility.
- Viral types: set to Influenza A and B co-circulation.
- Immunisation rate: set to High, indicating widespread vaccine coverage.

Under these conditions, the probability of a high ‘Influenza Epidemic’ increased to 76.7%, while the probability of a low epidemic decreased to 23.3%. Despite high immunisation coverage, the combination of severe global influenza activity, absence of control measures, peak seasonal timing, and co-circulation of other respiratory diseases, and the likely source of viruses from Southeast Asia significantly elevated the probability of an influenza epidemic in SEQ compared with the baseline scenario. This underscores the importance of integrated public health strategies beyond vaccination alone.

### 3.4. Comparison of Scenario Analysis Findings

Following Scenario 1, eight additional scenarios were simulated by modifying the probabilities of specific nodes to represent extreme or high conditions. The resulting probabilities of a high influenza epidemic across these scenarios are summarised in Table 1, with detailed node configurations presented in Supplementary Figures S1–S8 and Supplementary Table S2. The analysis highlights the complex interplay of epidemiological, environmental, and behavioural factors influencing influenza epidemic risk in Queensland.

The following are the key comparative insights based on the scenario analysis findings:

- High epidemic risk under severe conditions: All nine scenarios, characterised by a severe global influenza season (100%), a peak influenza season (100%), co-circulation of influenza A and B with other respiratory infections (such as RSV and COVID-19), absence of control measures, and elevated climate change impact (above RCP 2.6), resulted in significantly higher epidemic probabilities (ranging from 63% to 86%) compared to the baseline (50.7%). These findings underscore the dominant influence of these overarching risk factors.
- Moderate influence of source country: Virus origin showed a modest impact. Under similar high-risk conditions, Scenario 1 (SE Asian origin, 76.7%) yielded a slightly higher epidemic probability than Scenario 2 (European origin, 71.5%), suggesting moderate regional differences in strain transmissibility or severity.
- Effect of control measures: The presence of public health interventions notably reduced epidemic risk. Scenario 4, which included control measures set to Yes, showed a lower probability (63%) compared to Scenario 2 (71.5%), where such measures were absent, highlighting the mitigating effect.
- Impact of vaccination rate: Lower vaccination rates consistently elevated risk. Scenario 3 (low vaccination coverage) showed an increase in probability to 80.6% compared to Scenario 1 (high vaccination coverage, 76.7%). Similar trends were observed in Scenarios 6 and 9, reinforcing the critical role of vaccination, but the risk also depends on the status of other factors.
- Season duration: A longer influenza season (Scenario 6) increased epidemic probability (82%) compared to a shorter season (Scenario 2, 71.5%), indicating a moderate effect when controlling for other factors.
- Number of incoming travellers: A higher traveller volume significantly increased epidemic risk. Scenario 8, with low traveller numbers, showed a reduced probability (74.9%) compared to Scenario 6 (82%), under otherwise identical conditions. This aligns with seasonal travel patterns in Australia, particularly during school and public holidays and northern hemisphere summer vacations.
- Regional differences: SEQ appears slightly more vulnerable than North Qld. Scenario 6 (SEQ, 74.1%) showed a higher probability than Scenario 7 (North Qld, 71.5%), even after accounting for vaccination effects, suggesting regional susceptibility linked to population density and connectivity.

Table 1 summarises the comparative results of the simulated scenarios, focusing on variations in geographical characteristics and key risk factors (control measures, traveller volume, season duration, and vaccination coverage). Scenario 9 represents the worst-case scenario, with all key variables set to 100% in favour of high-risk conditions, resulting in the highest epidemic probability (86%).

### 3.5. Retrospective Validation Against Documented Epidemics

To evaluate real-world predictive validity, the model was retrospectively applied to three documented influenza epidemic periods in Queensland. Queensland Health surveillance reports confirmed intense influenza seasons during the years 2015, 2017, and 2019, with epidemic-level activity peaking in winter, consistent with the periods of July–September 2015, June–August 2017, and July–October 2019 [30]. For each documented epidemic, evidence on the conditions present during that period (season, global severity, regional factors, immunisation coverage, etc.) was entered into the model to assess whether it predicted an elevated epidemic risk. The results are shown in Table 2.

The model correctly identified an elevated epidemic risk (>65%) in all three documented epidemic periods, with two out of three exceeding 70% probability. The 2015 epidemic showed moderate-high probability (65.3%), potentially reflecting earlier seasonal timing and lower initial immunisation coverage that year. This retrospective validation supports the model’s ability to identify high-risk conditions corresponding to actual epidemic occurrence.

### 3.6. Sensitivity Analysis Results

Supplementary Figure S9 details a tornado diagram generated in GeNIe software. A tornado diagram is used to visualise a sensitivity analysis, demonstrating how changes in input variables affect the probability of a specific outcome, in this case, the target node representing the probability of an influenza epidemic.

The sensitivity analysis in this study was performed using the baseline scenario (Figure 1), where the probability of the target outcome (Influenza_Epidemic = High) is 0.507196, with a reachable range of ±10%: [0.502691, 0.5117]. This indicates the range of possible estimated probabilities for a high epidemic under the baseline network configuration, depending on variations in input variables. The diagram uses a red-to-green colour scheme where red portions represent parameter values that increase epidemic probability above the baseline, and green portions represent parameter values that decrease epidemic probability below the baseline.

Table 3 summarises a series of tornado diagram analyses from the Bayesian network model, showing how much each input variable or scenario affects the probability of a high influenza epidemic. The more a variable shifts the outcome probability from the baseline (0.507196), the higher its sensitivity ranking. The tornado diagram illustrates that the number of incoming travellers is by far the most influential variable affecting influenza epidemic probability, with the widest band in the visualisation. This indicates that travel-related interventions would have the greatest impact on epidemic prevention. A combination of the severity of a global influenza season and a long season duration is the second most impactful contributor to the high probability of an influenza epidemic. The analyses also provide additional information on the influences of population density (especially in SEQ) and control measures on the transmission of respiratory infections. Some climatic variables present weak or indirect influences, potentially due to model structure or uncertainty in links between climate and influenza transmission.

The narrow overall range of variation (0.5027 to 0.5117) from the baseline probability suggests that while these factors do influence epidemic probability, the model predicts a probability very close to 50%, with relatively modest variations based on the input parameters studied.

### 3.7. Model Evaluation Results

**Figure 3:**
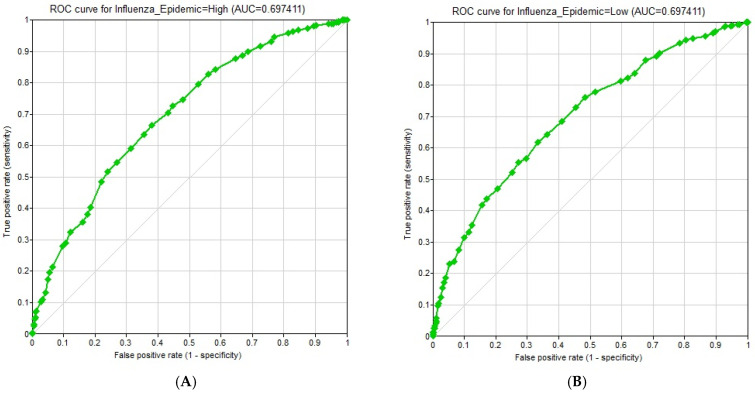
(**A**) ROC curve for influenza epidemic “High” (left), (**B**) ROC curve for influenza epidemic “Low” (right). The grey diagonal line indicates no better odds of detecting an influenza epidemic than random guessing; For a model to be useful, the green curve must stay above this diagonal line.

Figure 3A,B display the results of Receiver Operating Characteristic (ROC) curves for the outcome (“Influenza_Epidemic”) prediction, separated for the “High” and “Low” states. The ROC curve evaluates the model’s ability to distinguish between “Low” and “High” influenza epidemics. An area under the curve (AUC) of 0.5 suggests no discrimination (like random guessing), while an AUC of 1.0 represents perfect discrimination. The ROC curve (AUC = 0.6974) for the “High” state of influenza epidemic prediction indicates that the model has a fair to good ability to distinguish between the “High” and “Low” states. Interestingly, the ROC curve for Influenza_Epidemic = Low shows the same value (AUC = 0.6974). This is expected for a binary outcome classification when the ROC curve is generated for one class versus the other class.

Supplementary Table S2 shows a confusion matrix for the “Influenza_Epidemic” class node. A confusion matrix is a table that is often used to describe the performance of a classification model on a set of test data for which the true values are known. The overall accuracy of the model is about 70%. This means that the model correctly predicted the state of the influenza epidemic in 700 out of 1000 cases. The results also showed that the model correctly classified 636 out of 1000 (63.6%) “High”-class simulation instances (proportion of true positives = 354/503 ≈ 0.703777). On the other hand, the model accurately classified 282 out of 497 “Low”-class instances, resulting in a proportion of true negatives of 56.74% (282/497 ≈ 0.5674).

In summary, these figures and tables provide a comprehensive evaluation of the performance of the Bayesian network model in predicting influenza epidemics, including accuracy values, a confusion matrix, and ROC curves with AUC values for both “High” and “Low” epidemic states. The results suggest a reasonably capable model, with an overall accuracy of 70% and AUC values around 0.6974, indicating decent discriminative power.

## 4. Discussion

### 4.1. Key Findings

This study developed and validated a Bayesian network model for influenza epidemic risk assessment in Queensland, which demonstrated good discriminative performance (AUC = 0.6974, 70% accuracy). Scenario analyses revealed that Southeast Queensland faces the highest epidemic risk during peak season (June–August), particularly under conditions of severe global influenza activity, absent control measures, low immunisation coverage, and high international traveller volume. The sensitivity analysis identified incoming traveller volume as the strongest individual predictor, followed by global influenza season severity combined with long season duration, and population density in SEQ. Furthermore, the model successfully identified elevated epidemic risk in three previously documented epidemic periods (2015, 2017, and 2019) through retrospective validation, supporting its utility for prospective risk assessment.

The BN model created in this study portrays a complex interplay of factors influencing the probability of an influenza epidemic in Queensland and captures the epidemiological features of influenza transmission patterns across regions. The BN model and sensitivity analysis have revealed that SEQ regions with high population density are at greater risk of influenza epidemics compared to Central and North Queensland, particularly when a long-lasting and severe influenza season occurs in both northern and southern hemispheres. SEQ, including large cities such as Brisbane, Ipswich, Logan, and Gold Coast, is the most populous region in Queensland and dense populations in SEQ increase the likelihood of respiratory disease transmission. In addition, the concurrent circulation of influenza A and B, as well as co-circulation with other respiratory diseases such as RSV and COVID-19, further increases the probability of influenza outbreaks.

The results also found that viral strains originating from Southeast Asia are more likely to trigger epidemics in Queensland than those from Europe or other continents. Many populated Southeast (SE) Asian countries are popular travel and holiday destinations for Australians. These countries are situated in subtropical/tropical regions, where influenza exhibits either biannual or year-round seasonality patterns [3,10,51]. Influenza circulates more frequently in these countries, which increases the chance of introducing influenza viruses to Australia through human movement and air travel [42,52]. Importantly, SE Asian countries with dense populations are recognised as a critical reservoir for the emergence and global dissemination of novel influenza strains [10,53,54]. A large outbreak in SEQ can be triggered when local conditions favour rapid transmission (e.g., during cold winter months).

The risk of an influenza epidemic may also increase during prolonged influenza seasons (e.g., overlapping seasons across the northern and southern hemispheres), especially when both influenza A and B viruses are co-circulating. Influenza viruses frequently mutate through antigenic drift. Longer seasons allow these mutations to accumulate, which means that they are more likely to escape existing immunity in the population over time, and thus contribute to seasonal epidemics [42,55]. When influenza A and B circulate simultaneously over an extended period, the chance of viruses mixing increases. This may create an opportunity for antigenic shift, potentially resulting in a new virus strain to emerge. A novel virus with pandemic potential could cause a drastic outbreak similar to the 2009 H1N1 pandemic [42,55,56].

Prolonged influenza seasons may not only facilitate genetic changes within influenza viruses but also increase the likelihood of co-circulation with other respiratory pathogens, such as respiratory syncytial virus (RSV) and SARS-CoV-2 (COVID-19) viruses. This overlapping viral activity contributes to increasingly complex virus-virus interactions that vary depending on context and population, particularly in young children [57,58]. Such interactions can alter transmission patterns, interfere with immune responses, and make disease outcomes more unpredictable. As SARS-CoV-2 transitions to become endemic, the simultaneous circulation of multiple respiratory viruses may increase the risk of outbreaks, complicate clinical management, and challenge the effectiveness of public health interventions [59]. Our findings underscore the importance of ongoing surveillance to better understand and respond to evolving viral dynamics. In response to the need for scalable and adaptable surveillance systems post the COVID-19 pandemic, in 2023, Queensland Health expanded the statewide Acute Respiratory Infection Surveillance Reporting system (previously Notifiable Influenza Surveillance) to include RSV and COVID-19 notifications. This was in accordance with the Australian National Surveillance Plan for COVID-19, Influenza, and RSV to help guide public health responses and mitigate the impact of these diseases [30,60].

The results of the scenario and sensitivity analyses indicated that the presence of control measures and high influenza vaccination coverage were associated with reduced epidemic probability, supporting their protective effect. Our findings therefore suggest that non-pharmaceutical interventions (NPIs), including reducing incoming travellers, implementing public health control measures, and improving influenza vaccination coverage, can mitigate the risk of outbreaks [61,62]. This is consistent with studies demonstrating a significant decrease in influenza cases during the first two years of the COVID-19 pandemic when stringent NPIs were in effect [63,64,65]. However, due to strong social and economic impacts, the implementation of widespread NPIs, especially border controls and travel restrictions, is complex and requires careful consideration.

The sensitivity analysis findings also confirmed that certain climate conditions, including low temperature, humidity, and rainfall, contribute to influenza epidemics. In addition, long-term climate change may enhance influenza transmission in tropical/subtropical regions of Queensland. Previous studies suggest that ‘cold and dry’ conditions facilitate influenza seasonal epidemics in temperate regions, while the ‘humid-rainy’ conditions partially explain increased influenza activity during monsoon seasons in tropical and subtropical regions. It is noted that there is a large variation in influenza seasonality patterns in the tropics/subtropics, and the contributing climate drivers remain inconclusive [4,10,66]. Our findings, based on Queensland data (with a large proportion of influenza cases occurring in SEQ), reflect mainly the climatic conditions in winter epidemics. Although existing studies on the influence of climate change on influenza epidemics remain inconclusive, recent studies observed an increasing trend of inter-seasonal influenza in Australia over time [67,68]. This highlights the importance of continuing to monitor influenza epidemics in tropical and subtropical regions.

### 4.2. Applications of a Bayesian Network Model in Influenza Epidemics

Beyond quantifying epidemic risk, the developed BN model provides a structured, evidence-informed framework to support targeted intervention strategies. By ranking the influence of key variables, the model can be used to prioritise high-leverage points for outbreak prevention, such as traveller volume, vaccination coverage, or seasonal timing, enabling public health agencies to focus on the most effective control measures. This prioritisation is particularly valuable in resource-constrained settings, where early interventions targeting a few critical drivers can achieve substantial reductions in epidemic risk [28,29]. For example, directing vaccination campaigns towards regions with high population mobility and lower baseline immunity may yield greater population-level protection than uniform statewide approaches. Similarly, adjusting seasonal vaccination or communication strategies based on projected epidemic windows can improve program effectiveness while conserving public health resources [18,22].

The probabilistic nature of BN modelling makes it particularly well-suited to decision-making under uncertainty, where data may be incomplete, delayed, or inconsistent. Unlike deterministic models, BNs can infer missing data and still generate probabilistic forecasts when only partial information, such as mobility patterns, climatic conditions, or demographic profiles, is available [40,69]. The model can also be applied for diagnostic inference, as when an epidemic is observed, it can estimate posterior probabilities of likely causes, such as low vaccination coverage, a severe global influenza season, or the introduction of a new viral strain. This bidirectional reasoning enhances its value as an operational tool for both pre-season preparedness and real-time response planning during outbreaks [70,71].

The developed BN framework also provides a robust foundation for public health policy decision support. It can inform the optimal timing and targeting of vaccination campaigns by identifying periods and demographic groups at highest risk, support decisions around travel advisories and border controls by quantifying the effect of incoming travellers on local epidemic probabilities, and evaluate the potential outcomes of school closures or holiday policies by simulating changes in population mobility [14,72]. Its capacity to integrate climatic, demographic, and behavioural data makes it adaptable to emerging challenges such as altered seasonality patterns and long-term climatic shifts [4,10]. Consequently, the BN can serve as a flexible decision-making support tool for public health authorities in planning and implementing evidence-based influenza control strategies.

Furthermore, the model can be integrated with existing surveillance and monitoring systems to enhance situational awareness and early warning capabilities. Regular updates from meteorological datasets, public health surveillance (e.g., vaccination coverage, hospitalisations), and mobility data (e.g., transport networks, telecommunications) could be assimilated to continuously update epidemic forecasts [30,60]. This dynamic integration supports real-time epidemic intelligence and provides decision-makers with continuously refined estimates of regional and temporal influenza risk, allowing for adaptive policy and resource allocation in rapidly changing contexts.

Finally, one of the key advantages of Bayesian networks is their transparency and interpretability, which facilitate the communication of complex risk pathways to both technical and non-technical audiences. The visual and causal structure of the BN enables stakeholders to clearly understand how various drivers, such as seasonality, mobility, and immunity, interact to influence epidemic probability [37,40]. This visual traceability builds trust in the model’s logic and enhances collaborative policy dialogue across disciplines. By simplifying complex probabilistic dependencies into an accessible graphical format, the model strengthens evidence-based decision-making and supports the design of adaptive influenza preparedness strategies in Queensland and beyond.

### 4.3. Temporal Implementation and Real-Time Application

A critical consideration for operational use of the BN is how temporal dynamics are incorporated. The current BN represents a static “snapshot” of epidemic risk under specified conditions. However, temporal context is captured through:

- Seasonal variables: The “Season” node explicitly represents time of year, which is the primary temporal determinant of influenza risk in temperate and subtropical regions.
- Sequential updating: The model can be updated with new surveillance data on a weekly or bi-weekly cycle to provide time-varying risk estimates that reflect changing conditions.
- Rolling forecasts: By incorporating recent surveillance trends as evidence, the model can generate updated forecasts that implicitly account for recent epidemic trends.

Standard BNs do not explicitly model temporal autocorrelation, epidemic dynamics (exponential growth phases), or time-lagged relationships between variables. For example, the model does not inherently capture that high case numbers this week increase the probability of high cases next week due to transmission chains. Dynamic Bayesian networks (DBNs) extend static BNs by incorporating temporal dependencies between time slices, enabling explicit modelling of disease dynamics over time [73]. Future work will explore DBN implementations to capture the temporal evolution of epidemic risk and model multi-step forecasting horizons.

### 4.4. Limitations of the Model and Sources of Uncertainty

Despite the strengths of the Bayesian network model in integrating complex and heterogeneous data within an interpretable graphical framework, several limitations were identified in this study. These included:

- Temporal dynamics: The static BN does not explicitly model epidemic growth dynamics, temporal autocorrelation, or generation intervals. Consequently, the epidemic probability represents instantaneous risk under current conditions rather than the forward projection of case trajectories over multiple weeks.
- Independence assumptions: The BN assumes conditional independence given parent nodes. Some relationships may exhibit dependencies not captured by the current structure (e.g., complex interactions between climate variables beyond parent–child relationships).
- Simplified relationships: Continuous variables were discretised into categorical states, potentially losing information about non-linear relationships or threshold effects within categories.

### 4.5. Implications for Public Health Practice

This study highlights several key considerations for epidemic preparedness and public health planning in Queensland. Mobility is a major driver of influenza risk, with high volumes of incoming travellers, especially from Southeast Asia, substantially increasing the likelihood of outbreaks. This underscores the need for travel-related policies that are responsive to global influenza trends and adaptable to shifting movement patterns.

Vaccination coverage and non-pharmaceutical interventions (NPIs) remain vital. Even during severe seasons, timely measures such as mask use, physical distancing, and targeted immunisation can significantly reduce transmission. These strategies are particularly important in high-risk areas like Southeast Queensland, where population density and climate conditions favour viral spread.

Seasonal and climatic factors also play a critical role. Cold, dry winters and extended influenza seasons intensify transmission, and climate change may further disrupt established seasonal patterns, especially in tropical and subtropical zones. Public health strategies must evolve to reflect these changes.

Finally, the co-circulation of respiratory viruses, such as influenza, RSV, and COVID-19, complicates both clinical management and public health response. Integrated surveillance systems capable of detecting and responding to overlapping outbreaks are essential for maintaining healthcare system resilience and ensuring coordinated interventions.

### 4.6. Directions for Future Research

To improve the utility and accuracy of the Bayesian network (BN) model, future research should focus on several key areas. First, validating the model with real-time and multi-season data will be essential to confirm its reliability across diverse epidemiological settings. This will help ensure its robustness in forecasting outbreaks under varying conditions.

Second, investigating interactions between co-circulating viruses (e.g., influenza, RSV, and COVID-19) can offer deeper insights into transmission dynamics and inform more integrated intervention strategies. Understanding how these viruses influence each other may improve clinical and public health responses during overlapping outbreaks.

Third, incorporating high-resolution mobility and climate data will enhance the model’s precision and allow for more targeted, localised interventions. This will be particularly useful in adapting to rapidly changing environmental and behavioural conditions. Finally, assessing the scalability of the BN framework to other regions and respiratory pathogens will help determine its broader applicability. Expanding its use across different contexts can support national and international efforts to strengthen epidemic preparedness and response.

## 5. Conclusions

This study developed a Bayesian network model that integrated surveillance, climatic, demographic, and expert knowledge to estimate influenza epidemic probability in Queensland, Australia. The model demonstrated good discriminative performance (AUC = 0.6974, 70% accuracy) and successfully identified an elevated epidemic risk in previously documented epidemic periods through retrospective validation. Scenario analyses revealed that Southeast Queensland faces the highest risk of influenza epidemics during peak influenza season under conditions of severe global influenza activity, absent control measures, low immunisation coverage, and high international traveller volume, with the latter emerging as the strongest individual predictor.

The BN approach offers several distinct advantages for epidemic risk assessment, including the integration of heterogeneous data sources within a unified probabilistic framework, transparent visualization of causal relationships that is accessible to diverse stakeholders, natural handling of uncertainty and missing data through probability distributions, and scenario-based analysis capability without requiring extensive time-series data. These characteristics address key limitations of purely time-series or mechanistic modelling approaches and provide complementary decision-support functionality for epidemic preparedness.

Key limitations include the static nature of standard BNs, which do not explicitly model temporal epidemic dynamics or transmission chains, uncertainty associated with expert-elicited conditional probabilities, and limited historical training data, with only three large documented epidemics over a 10 year period. The narrow sensitivity range observed (0.503–0.512) reflects the modest influence of individual variables around the baseline scenario, whereas scenario analyses demonstrate that combinations of risk factors produce substantial probability increases (63–86%), highlighting the multifactorial nature of epidemic risk. Future directions incorporating dynamic Bayesian networks, expanded training data, and integration with mechanistic transmission models will enhance temporal forecasting capability and quantify structural uncertainty.

For practical implementation, the model provides a foundation for weekly epidemic risk updates that could be integrated with Queensland Health’s existing surveillance cycle through automated data feeds. Predefined risk probability thresholds could be used to trigger graduated public health responses, from enhanced surveillance (40–60%) to full epidemic activation (>75%). However, operational deployment would require the establishment of automated data pipelines, development of user-facing risk dashboards with clear interpretability features, training for health professionals in probabilistic interpretation, and ongoing model validation and refinement as new epidemic seasons are observed.

The broader significance of this work extends beyond Queensland and influenza specifically. The developed framework is adaptable to other jurisdictions and infectious diseases where multiple factors interact to determine outbreak risk, data is incomplete or uncertain, and decision-makers require interpretable and actionable guidance. As climate change alters disease seasonality patterns, novel pathogens emerge, and respiratory viruses co-circulate with increasing complexity, probabilistic modelling approaches that integrate diverse evidence sources will become increasingly important for epidemic preparedness and response.

This study demonstrates that Bayesian networks can provide valuable decision-support capability for influenza epidemic risk assessment when developed through stakeholder engagement, informed by empirical data and expert knowledge, validated against real epidemic events, and implemented with appropriate interpretation guidelines and uncertainty communication. The model offers Queensland Health a practical tool for enhancing epidemic preparedness, with potential application to other respiratory pathogens, such as RSV and COVID-19, and to regions facing similar forecasting challenges under uncertainty. By enabling targeted, evidence-based interventions rather than uniform population-wide approaches, such tools can optimise resource allocation and improve health outcomes in an era of evolving infectious disease threats.

## Figures and Tables

**Table 1:** Influenza epidemic risk comparison: Scenario analysis findings with uncertainty quantification.

Rank	Scenario	Epidemic Probability (95% CI)	Key Characteristics	Risk Factors
1	Scn 9	86.0% (81.2–90.1%)	SE Asian origin, SEQ	No control measures, Number of incoming travellers (High 100%), Long season, Low immunisation
2	Scn 6	82.0% (76.8–86.5%)	European origin, SEQ	No control measures, Number of incoming travellers (High 100%), Long season, Low immunisation
3	Scn 3	80.6% (75.1–85.4%)	SE Asian origin, SEQ	No control measures, Number of incoming travellers (High 100%), Short season, Low immunisation
4	Scn 1	76.7% (71.2–81.8%)	SE Asian origin, SEQ	No control measures, Number of incoming travellers (High 100%), Short season, High immunisation
5	Scn 8	74.9% (69.1–80.2%)	European origin, SEQ	No control measures, Number of incoming travellers (Low 100%), Long season, Low immunisation
6	Scn 7	74.1% (68.5–79.3%)	European origin, North Qld	No control measures, Number of incoming travellers (High 100%), Long season, High immunisation
7	Scn 2	71.5% (65.8–76.9%)	European origin, SEQ	No control measures, Number of incoming travellers (High 100%), Short season, High immunisation
8	Scn 5	64.4% (58.6–70.1%)	SE Asian origin, SEQ	Yes control measures, Number of incoming travellers (High 50%), Short season, High immunisation
9	Scn 4	63.0% (57.1–68.7%)	European origin, SEQ	Yes control measures, Number of incoming travellers (High 100%), Short season, High immunisation
10	Baseline	50.7% (47.2–54.3%)	Baseline	No certainties, balanced status across variables

Note: CI = Credible Interval. Other controlled conditions for Scenarios 1 to 9 include peak season (Jun–Aug), severe global influenza seasons, co-circulation of influenza A and B and other respiratory infections, and climate change scenario > RCP 2.6.

**Table 2:** Retrospective validation results.

Year	Epidemic Period	Region	Model-Predicted Probability (95% CI)	Observed Outcome
2015	Jul–Sep	SEQ	65.3% (58.2–72.1%)	Epidemic
2017	Jun–Aug	SEQ	73.8% (67.4–79.5%)	Epidemic
2019	Jul–Oct	Multiple	71.2% (64.8–77.3%)	Epidemic

**Table 3:** Top ten influential factors on influenza epidemic outcome (ranked by impact).

Rank	Variable Combination	Effect	Influence Type	Interpretation
1	Number of incoming travellers = High	Strongest (~50.3% to 51.2%)	Heavily Positive	High traveller volumes create a constant influx of diverse viral strains, challenging local containment.
2	Severity of influenza season in northern/southern hemisphere = Severe + Influenza season = Long	Very Strong (~50.4% to 51.0%)	Heavily Positive	When both hemispheres experience severe seasons with long durations (e.g., overlapping seasons), it facilitates high viral loads beyond the usual season.
3	Population density = High + Regions = SEQ	Strong (~50.4% to 50.9%)	Heavily Positive	Southeast Queensland’s high population density facilitates viral transmission through public transport, shopping centres, childcare centres, and workplaces.
4	Humidity = Low + Temperature = Below 20 °C + Rainfall = Low	Moderate-Strong (~50.5% to 50.9%)	Moderately Positive	Cool, dry conditions are in favour of influenza viral survival and transmission.
5	Control measures = No + Short season + No co-circulation + Mild severity	Moderate (~50.5% to 50.85%)	Positive	Absence of control measures is a key driver enabling the spread of influenza despite seemingly “mild” conditions.
6	Season = Dec–Feb	Moderate (~50.5% to 50.9%)	Moderately Negative	December-February (summer in Australia) acts as nature’s “viral circuit breaker”. High temperatures and humidity during summer suppress influenza activity.
7	Long season + Climate change > RCP 2.6 + North Qld	Low-Moderate (~50.5% to 50.85%)	Positive	Long-term climate change may disrupt traditional seasonal patterns, especially in tropical North Qld, making influenza transmission patterns more complex and unpredictable. With prolonged seasons in this tropical region, mild summer influenza clusters may trigger large outbreaks.
8	Long season + Climate change > RCP 2.6 + Central Qld	Low-Moderate (~50.6% to 50.9%)	Slightly Positive	Influenza seasonality patterns in Central Queensland are complex due to its mixed climate conditions (subtropical and some tropical). Under the influence of long-term climate change, this location may experience more unpredictable influenza transmission patterns, potentially extending traditional influenza seasons.
9	Season = June–Aug	Lower (~50.6% to 50.7%)	Weakly Positive	Expected seasonal influenza peaks coincide with winter months.
10	Source country = Others	Lowest (~50.6% to 50.7%)	Negative	The negative effect suggests these sources may indicate contained, traceable outbreaks rather than community spread.

## Data Availability

Data supporting specific components of the BN model development were sourced from publicly available databases, as detailed in Supplementary Table S1.

## Supplementary Materials

The following supporting information can be downloaded at: https://www.mdpi.com/article/10.3390/ijerph23010069/s1, Table S1: Variable definitions and data sources; Figure S1: Severe peak season epidemic in SEQ with high circulation, no control measures and high number of incoming travellers (European origin); Figure S2: Severe peak season epidemic in SEQ with high circulation, no controls, and low immunisation rate (SE Asian origin); Figure S3: Severe peak season epidemic in SEQ with high circulation, control measures in place, and high immunisation rate (European origin); Figure S4: Severe peak season epidemic in SEQ with high circulation, control measures in place, high immunisation rate and balanced number of incoming travellers (SE Asian origin); Figure S5: Severe peak season epidemic in SEQ with high circulation, no control measures, low immunisation rate, high number of incoming travellers and long influenza season duration (European origin); Figure S6: Severe peak season epidemic in North QLD with high immunisation rate, high number of incoming travellers, no control measures, and long influenza season duration (European origin); Figure S7: Severe peak season epidemic in SEQ with low immunisation rate, low number of incoming travellers, no control measures, and long influenza season duration (European origin); Figure S8: Severe peak season epidemic in SEQ with high circulation, no control measures, high number of incoming travellers, low immunisation rate and severe global season (SE Asian origin); Figure S9: Tornado diagram: Sensitivity analysis of the outcome node “Influenza Epidemic = High”; Table S2: Confusion matrix (model accuracy estimates).
