# Supplementary material for: Integrating Surveillance and Stakeholder Insights to Predict Influenza Epidemics: A Bayesian Network Study in Queensland, Australia
Source: Int J Environ Res Public Health. 2026 Jan 1;23(1):69. doi: 10.3390/ijerph23010069 (PMC12841570; doi:10.3390/ijerph23010069)
Supplement: Supplementary file 1 [file ijerph-23-00069-s001.zip › ijerph-4007103-supplementary.pdf]

Supplementary Document

Supplementary Table S1. Variable definitions and data sources.

Variable	Key Nodes/ Variables	Variable Description	Source	Data Availability/Comments
V1	Influenza epidemic	Number of weekly confirmed influenza cases reported to health authorities (2011-2022)	https://www.health.qld.gov.au/clinical-practice/guidelines-procedures/diseases-infection/surveillance/reports/flu https://www.health.gov.au/resources/collections/aisr	Data by state and Qld hospital and health service (HHS) available
V2	Source country	High risk countries/locations/ hemisphere identified by WHO FluNet	https://www.who.int/tools/flunet	
V3	Number of incoming travellers	Migrants, students, tourists, returning Australians (by Australian Bureau of Statistics (ABS))	https://www.abs.gov.au/statistics/industry/tourism-and-transport/overseas-arrivals-and-departures-australia/latest-release#:~:text=Key%20statistics,-September%202020%20original&text=Overseas%20visitor%20arrivals%20to%20Australia,previous%20month%20to%208%2C170%20trips	
V4	Immunity in population	Level of immunity based on age, immunisation and circulation with other respiratory diseases		

V5	Influenza immunisation rate	Overall national influenza immunisation rate in populations in the previous year/immunisation coverage	https://www.health.gov.au/topics/immunisation/immunisation-data/influenza-immunisation-data	
V6	Mobility	International travel and domestic travel (interstate travel and within each state)	https://www.abs.gov.au/statistics/industry/tourism-and-transport/overseas-arrivals-and-departures-australia/latest-release#:~:text=Key%20statistics,-September%202020%20original&text=Overseas%20visitor%20arrivals%20to%20Australia,previous%20month%20to%208%2C170%20trips<br>https://www.tourism.australia.com/en/markets-and-stats/tourism-statistics.html<br>http://www.regionalaustralia.org.au/home/regional-movers-index/<br>https://www.abs.gov.au/statistics/people/population/regional-internal-migration-estimates-provisional/latest-release	
V7	School/public holidays	Australian/Qld holidays	https://www.qld.gov.au/recreation/travel/holidays	Official calendar published each year
V8	Control measures for respiratory infections	Government COVID-19 control measures: Our World in Data (COVID-19 Stringency Index: Australia)	https://ourworldindata.org/explorers/covid?Metric=Stringency+Index&Interval=Daily&Relative+to+population=false	
V9	Co-circulations with other respiratory diseases	Including COVID-19 and RSV (Qld weekly surveillance reporting)	https://www.health.qld.gov.au/clinical-practice/guidelines-procedures/diseases-infection/surveillance/reports/flu	

V10	Influenza viral types	A/B cocirculation, A(H1N1), A(H3N2), B	https://www.health.qld.gov.au/__data/assets/pdf_file/0029/713675/influenza-qld-annual.pdf	Influenza A accounted for approximately 68%, and influenza B 32% according to Qld report 2013-18. The dominant viral type varies from year to year.
V11	Severity of influenza season in northern/ southern hemisphere	FluNet produces weekly updates and flags the high incidence regions	https://www.who.int/tools/flunet	Currently unavailable
V12	Gender	Gender ratio of Qld average reported cases 2013-2018	https://www.health.qld.gov.au/__data/assets/pdf_file/0029/713675/influenza-qld-annual.pdf	
V13	Age group	Age distribution of Qld average reported cases 2013-2018	https://www.health.qld.gov.au/__data/assets/pdf_file/0029/713675/influenza-qld-annual.pdf	
V14	Population density	ABS regional population report	https://www.abs.gov.au/statistics/people/population/regional-population/latest-release#queensland	
V15	Humidity	Weekly average absolute humidity/relative humidity (by Bureau of Meteorology (BOM))	https://www.bom.gov.au/climate/data/index.shtml	
V16	Temperature	Weekly average temperature (by BOM)	https://www.bom.gov.au/climate/data/index.shtml	

V17	Rainfall	Weekly average rainfall (by BOM)	https://www.bom.gov.au/climate/data/index.shtml	
V18	Influenza season duration	Defined as long or short compared to the average peak duration	Average duration in Queensland: 5 months (May - Sep)	Week 1-13 inter-seasonal; Weeks 14-22: beginning of season; Weeks 23-36: middle of the season; Weeks 37-44: end of season; 45-52: inter-seasonal
V19	Seasons (four seasons)	In the Southern Hemisphere: Mar-May (Autumn), Jun-Aug (Winter), Sep-Nov (Spring), Dec-Feb (Summer)		
V20	Climate change	RCP (Representative Concentration Pathway) 2.6 and ≥RCP 2.6 scenarios (global mean temperature increase <=2°C; > 2°C)	https://doi.org/10.1017/9781009157896.001	
V21	Region	Queensland Regions: North (Tropical), Central, Southeast Queensland (SEQ, Subtropical to Temperate)		

Supplementary Figures S1-8 Scenario analyses with result interpretation

Scenario 2 Severe Peak Season Epidemic in SEQ with European Origin, High Circulation, No Controls and High Number of Incoming Travellers

In this second scenario, we observe a similar setup to Scenario 1, with most of the key influencing factors retaining their 100% probability states. The only change in this scenario is 'European origin' of influenza instead of SE Asia, as depicted in Supplementary Figure S1.

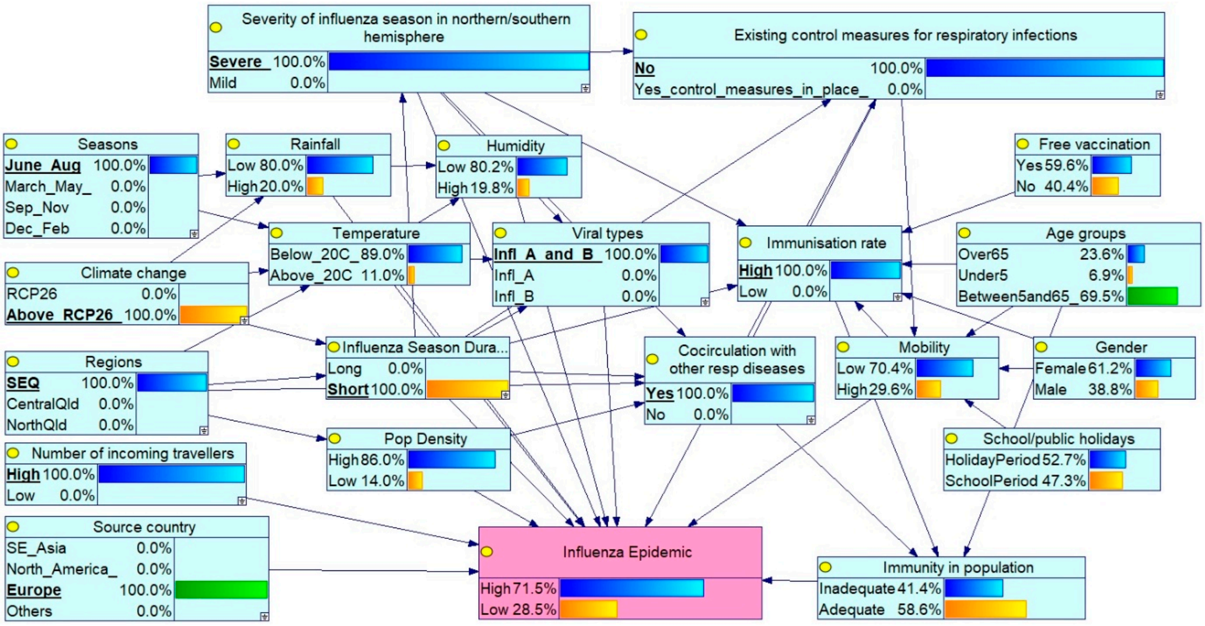

Supplementary Figure S1. Severe peak season epidemic in SEQ with high circulation, no control measures and high number of incoming travellers (European origin).

As a result of influenza origin, the probability of an influenza epidemic being high is now 71.5%, and the probability of it being Low is 28.5%. Comparing this outcome to Scenario 1 (where the probability of a high epidemic was 76.7% with the source country being SE Asia), we observe a slight decrease in the predicted probability of a high chance of influenza epidemic when the source of the virus is Europe instead of SE Asia, under the same specific circumstances.

This suggests that, within this model and given the other conditions, the origin of the introduced virus might have a marginal impact on the overall epidemic risk in SEQ. The model parameters or the specific probabilities associated with the "Source Country" node may indicate a slightly lower risk of a severe epidemic when the initial introduction is from Europe compared to SE Asia. However, the probability of a high influenza epidemic remains high (71.5%) due to the combination of other high-risk factors.

Scenario 3 Severe Peak Season Epidemic in SEQ with SE Asian Origin, High Circulation, No Controls, and Low Immunisation Rate

In this third scenario, depicted in Supplementary Figure S2, we see a similar high-risk setup as in Scenario 1, but with a key change in influenza immunisation rate (changed from high to low).

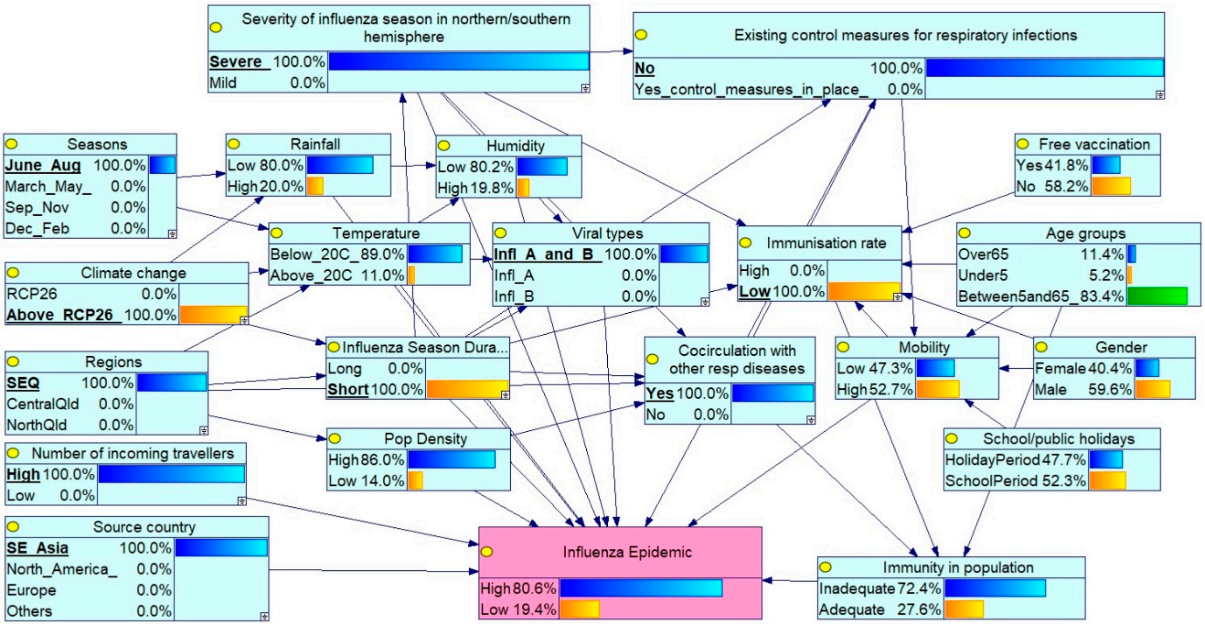

Supplementary Figure S2. Severe peak season epidemic in SEQ with high circulation, no controls, and low immunisation rate (SE Asian origin).

As a result of the change in influenza vaccination from extremely high to extremely low, the probability of an influenza epidemic being high increases to 80.6%, and the probability of it being low is 19.4%. This suggests that, within this model, SEQ has an elevated risk of a high epidemic under similar conditions compared to Scenario 1, but with a change in immunisation.

Scenario 4 Severe Peak Season Epidemic in SEQ with European Origin, High Circulation, High Immunisation Rate, and High Level of Control Measures

In this scenario, shown in Supplementary Figure S3**,** all conditions are kept the same as Scenario 2, except 'control measures' (changed from '100% No' to '100% Yes').

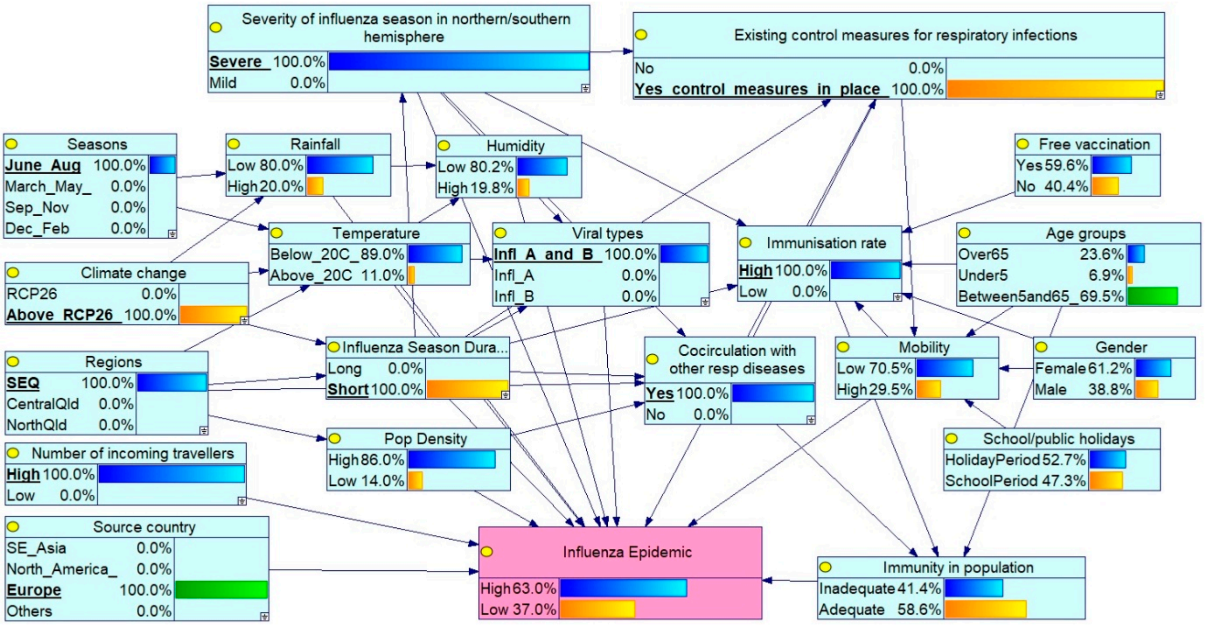

Supplementary Figure S3. Severe peak season epidemic in SEQ with high circulation, control measures in place, and high immunisation rate (European origin).

Compared with Scenario 2, the change in 'control measures' from 'No' status to 'Yes' has reduced the probability of a high influenza epidemic from 71.5% to 63.0%. This demonstrates the effect of rigorous public health control measures in place (targeting respiratory infections) on the reduction of epidemic probability despite severe conditions in favour of influenza transmission.

Scenario 5 Severe Peak Season Epidemic in SEQ with SE Asian Origin, High Circulation, High Immunisation Rate, Medium Level of Incoming Travellers and High Level of Control Measures

This scenario, depicted in Supplementary Figure S4, simulates severe conditions similar to Scenario 1 but with control measures in place and a reduction in the number of incoming/overseas travellers (from 100% to 50%). The overall probability of a high influenza epidemic is now at 64.4%, a significant reduction from Scenario 1 (probability of high influenza epidemic: 76.7%). This result suggests that reduced population mobility, especially a reduced volume of incoming travellers, and having control measures in place can largely reduce the probability of an influenza epidemic.

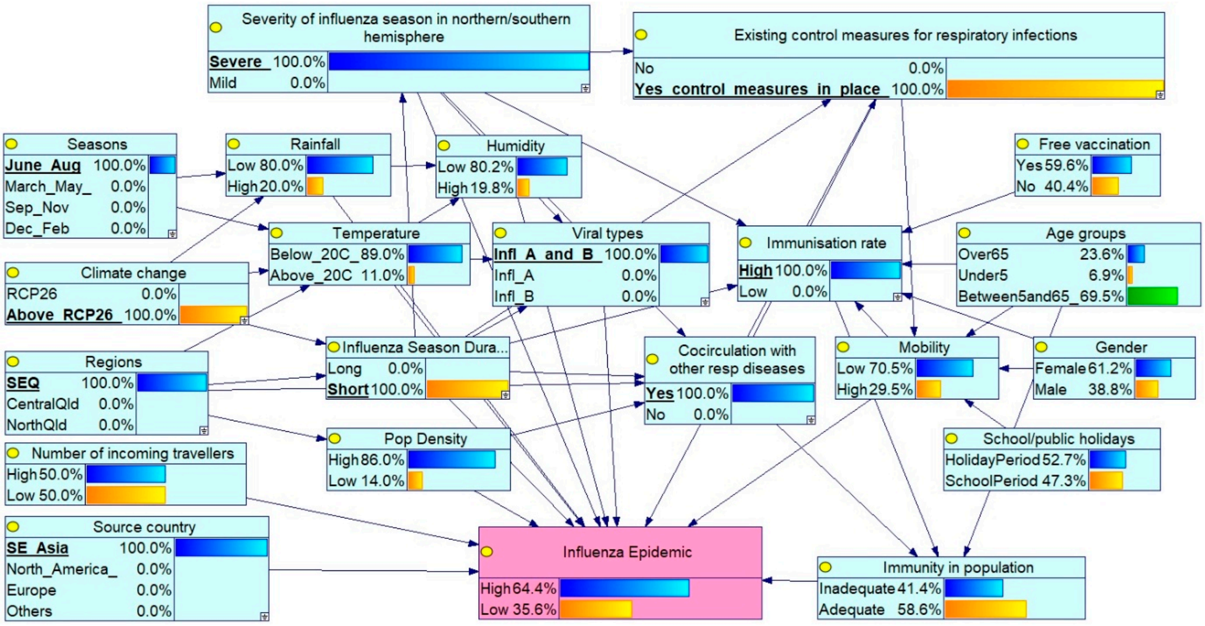

Supplementary Figure S4. Severe peak season epidemic in SEQ with high circulation, control measures in place, high immunisation rate and balanced number of incoming travellers (SE Asian origin).

Scenario 6 Severe Peak Season Epidemic in SEQ with European Origin, High Circulation, Low Immunisation Rate, High Number of Incoming Travellers, No Control Measures, and Long Influenza Season Duration

Scenario 6, shown in Supplementary Figure S5, represents a high-risk configuration with an 82.0% high risk of an Influenza Epidemic. The high risk is primarily driven by long influenza season (100%), the absence of control measures, high incoming travellers (100%) and peak seasonal timing during June-August. Compared with Scenario 2, the long seasonal duration and low influenza immunisation increase the risk of transmission. Although European origin viruses have been shown in other scenarios to pose slightly lower risk than those from Southeast Asia, this scenario illustrates that origin becomes a less influential factor when other domestic conditions (no control measures, low immunisation and high number of travellers) are highly conducive to spread. This highlights the importance of control measures.

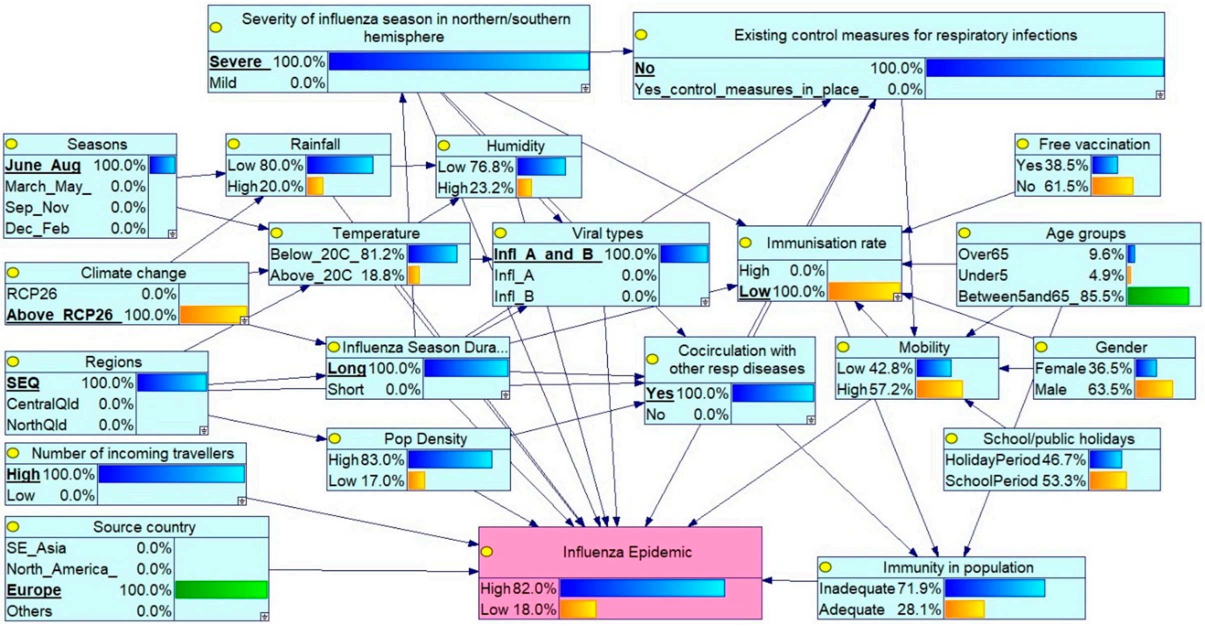

Supplementary Figure S5. Severe peak season epidemic in SEQ with high circulation, no control measures, low immunisation rate, high number of incoming travellers and long influenza season duration (European origin).

Scenario 7 Severe Peak Season Epidemic in North Qld with European Origin, High Circulation, High Immunisation Rate, High Number of Incoming Travellers, No Control Measures, and Long Influenza Season Duration

Scenario 7, shown in Supplementary Figure S6, follows similar conditions as seen in Scenario 6, apart from the location being changed to North Qld and the immunisation rate being high (100%). The probability of a high-risk influenza epidemic reduces to 74.1%. This scenario shows the effects of lower intensity of influenza epidemic during the peak season (June - August) in tropical Qld compared with SEQ and high-level vaccination.

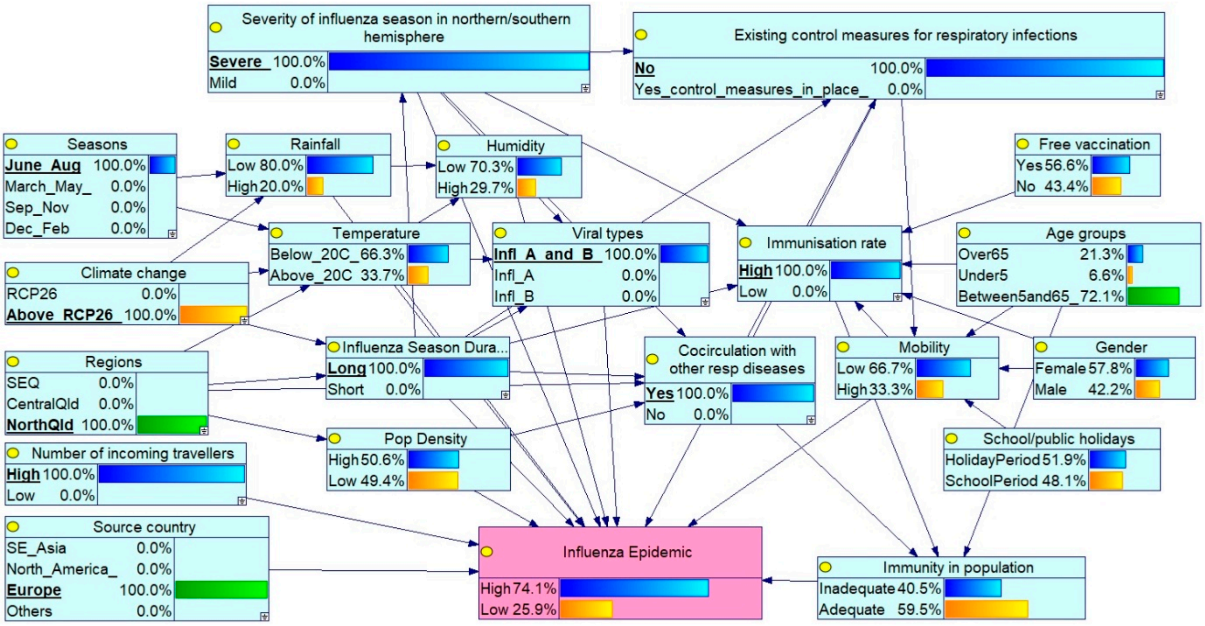

Supplementary Figure S6. Severe peak season epidemic in North QLD with high immunisation rate, high number of incoming travellers, no control measures, and long influenza season duration (European origin).

Scenario 8 Severe Peak Season Epidemic in SEQ with European Origin, High Circulation, Low Immunisation Rate, Low Number of Incoming Travellers, No Control Measures, and Long Influenza Season Duration

Scenario 8, represented in Supplementary Figure S7, keeps all conditions as in Scenario 6, except changing 'the number of incoming travellers' from extremely high to extremely low (100%). This difference has changed the 'high chance' of influenza epidemic from 82.0% to 74.9%, highlighting the impact of influx of travellers from overseas or interstate (a high level of population mobility).

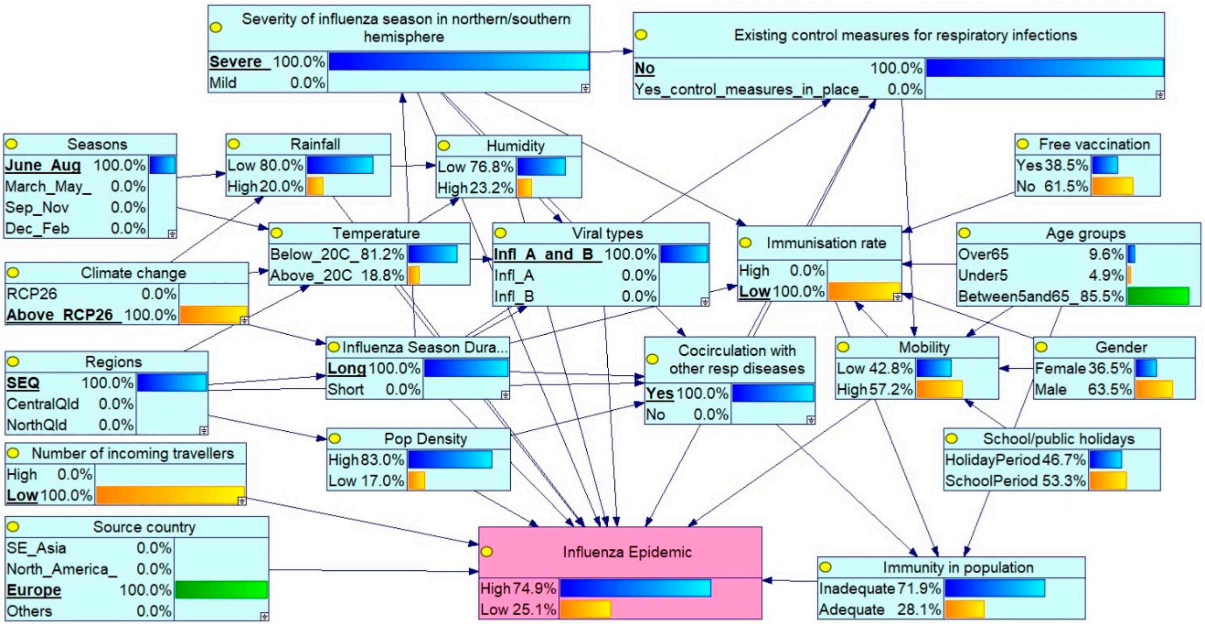

Supplementary Figure S7. Severe peak season epidemic in SEQ with low immunisation rate, low number of incoming travellers, no control measures, and long influenza season duration (European origin).

Scenario 9 Severe Peak Season Epidemic in SEQ with SE Asian Origin, High Circulation, Low Immunisation Rate, High Number of Incoming Travellers, No Control Measures, Long Influenza Season Duration and Severe Global Season

Scenario 9, depicted in Supplementary Figure S8, demonstrates the 'worst case scenario' with all the aforementioned conditions set extremely high (100%), leading to a very high probability of the target node (influenza epidemic) at 86.0%. Compared to the second highest-risk scenario (Scenario 6 = 82.0%), the virus source country shifting to SE Asia has contributed to the higher probability of influenza epidemic in this simulation.

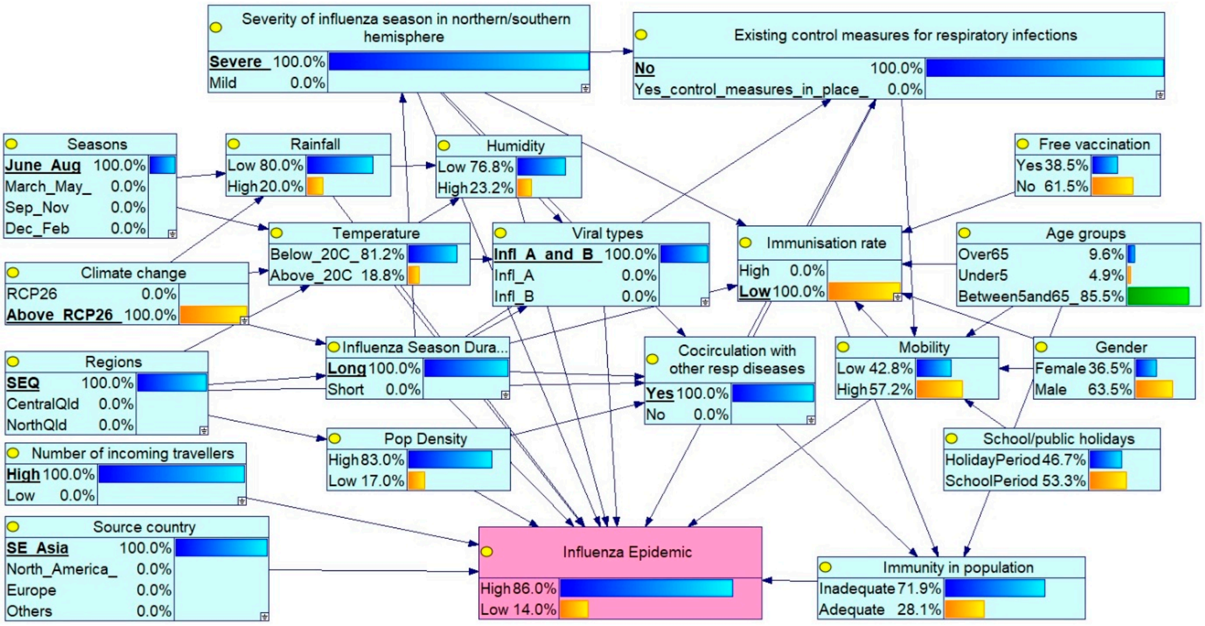

Supplementary Figure S8. Severe peak season epidemic in SEQ with high circulation, no control measures, high number of incoming travellers, low immunisation rate and severe global season (SE Asian origin).

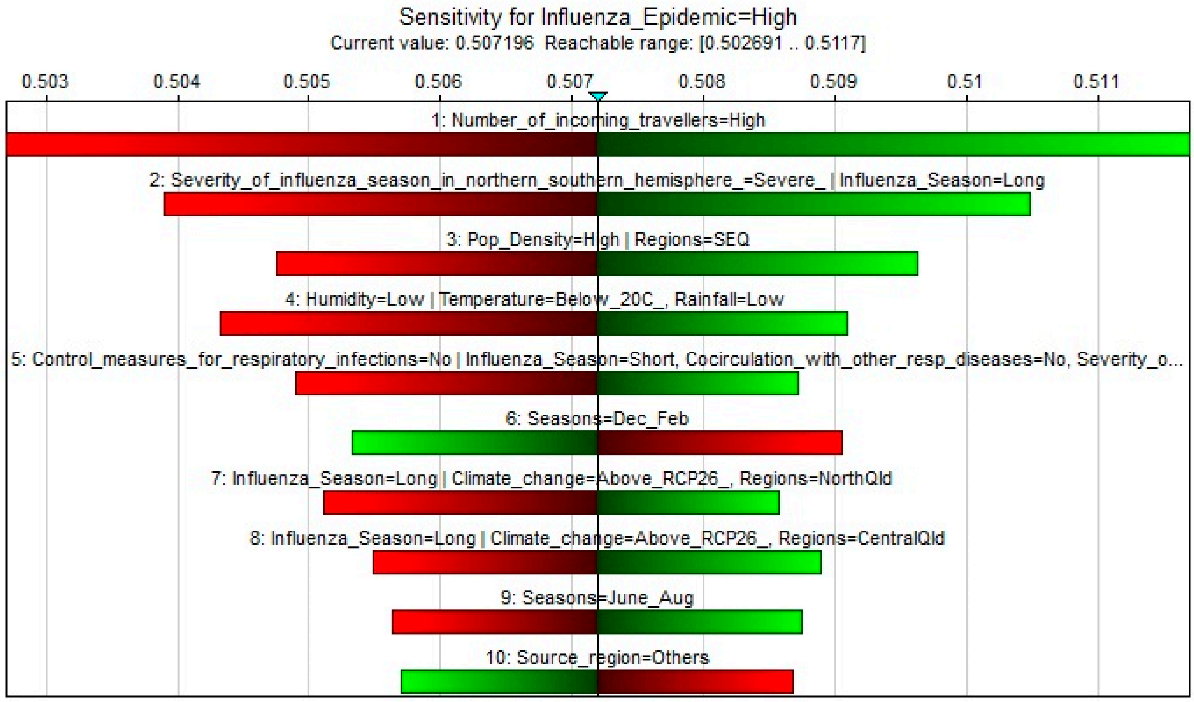

Supplementary Figure S9. Tornado diagram: Sensitivity analysis of the outcome node "*Influenza Epidemic = High*".

Notes:

- Bars: Each bar shows how much a variable can push the probability of the outcome up or down.
- Bar Length: The longer the bar, the greater the influence that variable has.
- Green/Red:
  - Green: Value combinations that increase the outcome probability.
  - Red: Value combinations that decrease the outcome probability.
  - Sorted Top-Down: From most to least influential variables.

The tornado diagram reports a sensitivity analysis that orders model parameters by their impact on the probability of "Influenza Epidemic = High." Bar length reflects the width of the target value range, so "Number_of_incoming_travellers = High" has the greatest influence, with its parameter range producing the largest change in epidemic probability and therefore appearing at the top of the plot.

For each parameter, the derivative represents the local rate of change in the outcome probability with respect to that parameter; a positive derivative indicates that increasing the parameter raises epidemic risk, whereas a negative derivative indicates a risk-reducing effect. Parameters describing severe and prolonged influenza seasons in source regions, high population density in SEQ, adverse meteorological conditions, winter circulation, and climate-change–amplified transmission in North and Central Queensland all show positive derivatives but shorter bars, implying secondary influence

relative to traveller volume. By contrast, configurations such as “Seasons = Dec_Feb” and “Source_region = Others” display negative derivatives and narrow ranges, suggesting that shifting probability mass toward these states slightly decreases the modelled probability of a high influenza epidemic.

The numerical details of the 10 Tornado diagram analyses in Supplementary Figure S9 are recorded below.

1. Number_of_incoming_travellers=High

Target value range: [0.502691, 0.5117], width=0.00900902
Parameter range: [0.45 . 0.55], width=0.1
Current parameter value: 0.5 at definition index 0
Derivative: 0.0900902
Coeffs: a=0.0900902, b=0.46215, c=2.66454e-15, d=1

2. Severity_of_influenza_season_in_northern_southern_hemisphere_=Severe; Influenza_Season=Long

Target value range: [0.503899, 0.510492], width=0.00659385
Parameter range: [0.72, 0.88], width=0.16
Current parameter value: 0.8 at definition index 0
Derivative: 0.0412115
Coeffs: a=0.0412115, b=0.474226, c=-5.55112e-16, d=1

3. Pop_Density=High; Regions=SEQ

Target value range: [0.504757, 0.509634], width=0.00487684
Parameter range: [0.72, 0.88], width=0.16
Current parameter value: 0.8 at definition index 0
Derivative: 0.0304803
Coefficients: a=0.0304803, b=0.482811, c=-2.77556e-16, d=1

4. Humidity=Low; Temperature=Below_20C; Rainfall=Low

Target value range: [0.504324, 0.50911], width=0.00478591
Parameter range: [0.84375, 1], width=0.15625
Current parameter value: 0.9375 at definition index 0
Derivative: 0.0306298
Coefficients: a=0.0306298, b=0.47848, c=-1.16573e-15, d=1

5. Control_measures_for_respiratory_infections=No; Influenza_Season=Short; Cocirculation_with_other_resp_diseases=No; Severity_of_influenza_season_in_northern_southern_hemisphere=Mild

Target value range: [0.504902, 0.508725], width=0.00382259
Parameter range: [0.84375, 1], width=0.15625
Current parameter value: 0.9375 at definition index 14
Derivative: 0.0244646
Coefficients: a=0.0244646, b=0.48426, c=2.22045e-16, d=1

6. Seasons=Dec_Feb

Target value range: [0.509066, 0.505325], width=0.00374112
Parameter range: [0.225, 0.275], width=0.05
Current parameter value: 0.25 at definition index 3
Derivative: -0.0748224
Coefficients: a=-0.0748224, b=0.525901, c=0, d=1

7. Influenza_Season=Long; Climate_change=Above_RCP26; Regions=NorthQld

Target value range: [0.505116, 0.508582], width=0.0034659
Parameter range: [0.84375, 1], width=0.15625
Current parameter value: 0.9375 at definition index 10
Derivative: 0.0221818
Coefficients: a=0.0221818, b=0.4864, c=-2.498e-16, d=1

8. Influenza_Season=Long | Climate_change=Above_RCP26_, Regions=CentralQld

Target value range: [0.505489, 0.508902], width=0.00341365
Parameter range: [0.69264, 0.84656], width=0.15392
Current parameter value: 0.7696 at definition index 8
Derivative: 0.0221781
Coefficients: a=0.0221781, b=0.490127, c=5.55112e-17, d=1

9. Seasons=June_Aug

Target value range: [0.505639, 0.508752], width=0.00311224
Parameter range: [0.225, 0.275], width=0.05
Current parameter value: 0.25 at definition index 0
Derivative: 0.0622448
Coefficients: a=0.0622448, b=0.491634, c=0, d=1

10. Source_region=Others

Target value range: [0.508694, 0.505697], width=0.00299677
Parameter range: [0.225, 0.275], width=0.05
Current parameter value: 0.25 at definition index 3
Derivative: -0.0599354
Coefficients: a=-0.0599354, b=0.522179, c=1.11022e-15, d=1

Supplementary Table S2. Confusion matrix (model accuracy estimates).

		Predicted		Accuracy
		High	Low	Influenza Epidemic = 0.636 (636/1000)
Actual	High	354	149	High = 0.703777 (354/503)
	Low	215	282	Low = 0.567404 (282/497)

Class node: Influenza Epidemic: This indicates that the model is trying to classify the state of an influenza epidemic.

- Actual (Act): This row represents the true states of the influenza epidemic (High or Low).
- Predicted: This column represents the states predicted by the model (High or Low).
- True Positives (TP) - Actual: High, Predicted: High (32): The model correctly predicted 32 instances where the influenza epidemic was High.
- False Negatives (FN) - Actual: High, Predicted: Low (17): The model incorrectly predicted 17 instances where the influenza epidemic was High as Low.
- False Positives (FP) - Actual: Low, Predicted: High (13): The model incorrectly predicted 13 instances where the influenza epidemic was Low as High.
- True Negatives (TN) - Actual: Low, Predicted: Low (38): The model correctly predicted 38 instances where the influenza epidemic was Low.

This table provides the accuracy of the model, broken down by overall and by each class.

- Accuracy: Influenza Epidemic = 0.7 (70/100): This is the overall accuracy of the model. It means that the model correctly predicted the state of the influenza epidemic in 70 out of 100 cases (which is the sum of True Positives and True Negatives divided by the total number of instances: (32+38) / (32+17+13+38) = 70/100 = 0.7).
- High = 0.653061 (32/49): This is the accuracy specifically for the "High" class. It's calculated as True Positives for "High" divided by the total actual "High" instances (32 / (32 + 17) = 32/49 ≈ 0.653). This is also known as the recall or sensitivity for the "High" class.
- Low = 0.745098 (38/51): This is the accuracy specifically for the "Low" class. It's calculated as True Negatives for "Low" divided by the total actual "Low" instances (38 / (13 + 38) = 38/51 ≈ 0.745). This is also known as the recall or sensitivity for the "Low" class.
